# Role of Human Antigen R (HuR) in the Regulation of Pulmonary ACE2 Expression

**DOI:** 10.3390/cells11010022

**Published:** 2021-12-22

**Authors:** Noof Aloufi, Zahraa Haidar, Jun Ding, Parameswaran Nair, Andrea Benedetti, David H. Eidelman, Imed-Eddine Gallouzi, Sergio Di Marco, Sabah N. Hussain, Carolyn J. Baglole

**Affiliations:** 1 Meakins-Christie Laboratories, Research Institute of the McGill University Health Centre (RI-MUHC), Montreal, QC H4A 3J1, Canada; Noof.Aloufi@mail.mcgill.ca (N.A.); zahraa.haidar@mail.mcgill.ca (Z.H.); Jun.ding@mcgill.ca (J.D.); david.h.eidelman@mcgill.ca (D.H.E.); sabah.hussain@mcgill.ca (S.N.H.); 2 Translational Research in Respiratory Diseases Program, Research Institute of the McGill University Health Centre, Montreal, QC H4A 3J1, Canada; andrea.benedetti@mcgill.ca; 3 Departments of Pathology, McGill University, Montreal, QC H3A 2B4, Canada; 4 Department of Medical Laboratory Technology, Taibah University, Universities Road, Medina 42353, Saudi Arabia; 5 Department of Medicine, McGill University, Montreal, QC H4A 3J1, Canada; 6 Department of Medicine, McMaster University & St Joseph’s Healthcare, Hamilton, ON L8N 4A6, Canada; parames@mcmaster.ca; 7 Department of Epidemiology, Biostatistics & Occupational Health, McGill University, Montreal, QC H3A 1G1, Canada; 8 Department of Biochemistry, McGill University, Montreal, QC H3G 1Y6, Canada; imed.gallouzi@mcgill.ca (I.-E.G.); sergio.di_marco@mcgill.ca (S.D.M.); 9 Smart-Health Initiative and Biological and Environmental Science and Engineering (BESE) Division, King Abdullah University of Science and Technology (KAUST), Thuwal, Jeddah 23955, Saudi Arabia; 10 Department of Pharmacology and Therapeutics, McGill University, Montreal, QC H3G 1Y6, Canada

**Keywords:** HuR, ACE2, fibroblast, COVID-19, chronic obstructive pulmonary disease, *ELAVL1*

## Abstract

Patients with COPD may be at an increased risk for severe illness from COVID-19 because of ACE2 upregulation, the entry receptor for SARS-CoV-2. Chronic exposure to cigarette smoke, the main risk factor for COPD, increases pulmonary ACE2. How ACE2 expression is controlled is not known but may involve HuR, an RNA binding protein that increases protein expression by stabilizing mRNA. We hypothesized that HuR would increase ACE2 protein expression. We analyzed scRNA-seq data to profile *ELAVL1* expression in distinct respiratory cell populations in COVID-19 and COPD patients. HuR expression and cellular localization was evaluated in COPD lung tissue by multiplex immunohistochemistry and in human lung cells by imaging flow cytometry. The regulation of ACE2 expression was evaluated using siRNA-mediated knockdown of HuR. There is a significant positive correlation between *ELAVL1* and *ACE2* in COPD cells. HuR cytoplasmic localization is higher in smoker and COPD lung tissue; there were also higher levels of cleaved HuR (CP-1). HuR binds to *ACE2* mRNA but knockdown of HuR does not change ACE2 protein levels in primary human lung fibroblasts (HLFs). Our work is the first to investigate the association between ACE2 and HuR. Further investigation is needed to understand the mechanistic underpinning behind the regulation of ACE2 expression.

## 1. Introduction

Chronic obstructive pulmonary disease (COPD) is a leading cause of morbidity and mortality worldwide [1]. COPD is now the third leading cause of death [2], which is expected to further increase in the coming decades. COPD is an umbrella term encompassing emphysema, which is the irreversible destruction of alveolar sacs, and chronic bronchitis that is characterized by a productive cough accompanied by an abnormal inflammatory response in the airways and lungs [3]. Smoking is the main risk factor for developing COPD [4], and people with COPD may also face an increased risk for severe illness from coronavirus disease (COVID-19) with increased hospitalization and intensive care unit (ICU) admission [5]. COVID-19 has rapidly spread through the world and is caused by severe acute respiratory syndrome coronavirus 2 (SARS-CoV-2), a novel β-coronavirus [6].

One reason that individuals with COPD may be at heightened risk for severe COVID-19 is the increased expression of the angiotensin-converting enzyme 2 (ACE2), the entry receptor for SARS-CoV-2 [6,7]. ACE2 is expressed in many organs, including the kidney, heart, and lungs [8]. Pulmonary ACE2 is concentrated mainly in type II alveolar cells and macrophages but is also present in bronchial and tracheal epithelial cells and lung fibroblasts [8,9,10,11,12]. Although ACE2 is the primary means for SARS-CoV-2 entry, maintenance of ACE2 levels is essential for combatting inflammatory and fibrotic lung disease [13]. ACE2 is part of the renin–angiotensin system (RAS) that cleaves angiotensin II to Ang-(1-7) [14]. Ang-(1-7) then activates the Mas receptor, leading to release of nitric oxide, prostaglandin E_2_, and bradykinin [15], resulting in vasodilation, natriuresis and a decrease in inflammation [16,17].

Therefore, changes in the expression of ACE2 may contribute to susceptibility to COVID-19 or predispose to post-COVID-19 complications. Environmental factors, such as smoking, increase ACE2 expression [18,19]. In COPD, ACE2 is overexpressed in alveolar and bronchial epithelium [20], and our group recently showed that COPD-derived lung fibroblasts express higher levels of ACE2; chronic cigarette smoke also increased ACE2 protein in mouse lungs [9]. Although ACE2 expression may be controlled at multiple levels (e.g., transcriptional, post-transcriptional and/or translational) [21], with the stability of mRNA factoring predominantly in the overall regulation of gene expression in response to changing environmental conditions (e.g., oxidative stress, hypoxia) [22], *ACE2* expression may be controlled by alterations in mRNA stability [23]. However, it is not well understood how ACE2 expression is regulated.

mRNA stability can be controlled by RNA-binding proteins (RBPs), such as human antigen R (HuR). HuR, encoded by the *ELAVL1* gene, belongs to the embryonic lethal abnormal vision (ELAV) family of RBPs [24,25]. HuR binds to mRNAs to control stability and/or translation of target mRNAs, and participates in pre-mRNA splicing and the nucleocytoplasmic translocation of target mRNAs [24,25,26]. These target mRNAs encode proteins involved in cellular processes, such as proliferation, differentiation, apoptosis, and inflammation [24,25]. HuR is mainly located in the nucleus in resting cells and translocates to the cytoplasm along with bound mRNA in response to stimuli, such as UVB, radiation, and cigarette smoke [24,25,27,28]. Although HuR is expressed in several lung cell types, including epithelial cells and fibroblasts [28], little is known about its expression in other lung cells. It is also not known whether HuR/*ELAVL1* expression changes in the context of lung disease, including COVID-19 and COPD, or whether the upregulation of ACE2 in smokers and COPD is regulated by HuR. Therefore, we hypothesis that HuR controls the expression of pulmonary ACE2.

Using scRNA-seq datasets, we first profiled the differential expression of *ELAVL1* in cells of the upper and lower respiratory system in COPD and COVID-19 subjects. We found a significant positive correlation between *ELAVL1* and *ACE2* in COPD cells, and that there is elevated cytoplasmic HuR protein in cells within smoker and COPD lungs. Mechanistically though, HuR does not control the upregulation of ACE2 in smoker and COPD cells despite HuR binding to the *ACE2* transcript. These data highlight the need for more mechanistic research into factors controlling pulmonary ACE2 expression. Identification of these mechanisms could lead to new therapeutic targets for both inflammatory and fibrotic stimuli as well as for SARS-CoV-2 entry into the respiratory system.

## 2. Materials and Methods

### 2.1. Chemicals

All chemicals were obtained from Sigma (St. Louis, MO, USA) unless otherwise indicated. Actinomycin D (ActD) was from Enzo Life Sciences.

### 2.2. Single Cell RNA-Sequencing Analysis

Raw single-cell RNA-seq expression matrices for lung cells [29] or nasopharyngeal/bronchial cells [30] were filtered using SCANPY software [31]. Cells with less than 200 expressing genes or more than 40% of mitochondrial reads were filtered. Genes that were only expressed in less than three cells were also removed. Genes were further filtered if they exhibited low dispersion (<0.15) or very low-level expression (<0.0125). The filtered gene expression matrix was then converted to log2 space ($\log_{2}\;expression+1$) for the following analysis. We then cluster cells from the obtained cell-by-gene expression matrices (by rows). The cell type annotations for the resulting clusters were directly downloaded from the original studies [29,30], which were based on known cell type markers. We further explored the expression of *ELAVL1* gene in various cell populations. Mann–Whitney U tests were used to quantify the difference in *ELAVL1* gene expression between different cell populations. The *ELAVL1* positive cells in each cell population (cluster) are also counted, and we sort all the cell types based on the percentage of *ELAVL1* positive cells. By comparing the difference in the percentage of *ELAVL1* positive cells, we can also identify a list of cell types with the most differential *ELAVL1* gene expression between conditions (e.g., COPD vs. control).

### 2.3. COPD Subjects

The study population included current smokers with COPD (COPD), smokers without COPD (smoker) and non-smokers without COPD (non-smoker; normal). Subject characteristics are as we have published [32]. Lung tissue was obtained from subjects undergoing lung resection surgery at McMaster University.

### 2.4. Multiplex Immunohistochemistry (mIHC)

mIHC was performed on lung tissues from non-smokers (control; M/F = 1/3; age 64.75 ± 6.1 years), current smokers with COPD (COPD; M/F = 1/3; age 54.5 ± 3.4 years) and without COPD (smoker; M/F = 0/3; age 67.3 ± 4.98 years) by the Discovery Ultra Ventana automated slide preparation system (Roche, Laval, QC, Canada). Briefly, formalin-fixed paraffin-embedded blocks were cut to a one 4-μm-thick section. Slides were deparaffinized at 69 °C and pretreated with CC1 (EDTA) for 24 min at 95 °C. Then, Discovery Inhibitor was added for 4 min. The slides were incubated with primary anti-HuR antibody (1:100; Santa Cruz, Dallas, TX, USA) for 15 min at 37 °C. Secondary antibody conjugated to horseradish peroxidase (HRP) (OMap anti-mouse HRP, Roche, Laval, QC, Canada) was incubated for 16 min. Immunodetection was performed with 3,3′-diaminobenzidine (DAB, Roche, Laval, QC, Canada). Then, primary anti-vimentin antibody (1:50; Cell Signaling Technologies, Whitby, ON, Canada) was incubated for 16 min at 37 °C followed by the secondary antibody UltraMap (anti- rabbit alkaline phosphatase) and incubated for 16 min at 37 °C. Immunodetection was performed with DISCOVERY Yellow kit. Next, the slides were incubated with the triple stain using the primary antibody for cytokeratin 19 (Roche, Laval, QC, Canada) for 16 min at 37 °C. The secondary antibody was multimer HRP (OMap anti-mouse HRP, Roche, Laval, QC, Canada) and was incubated for 16 min. Immunodetection was performed with DISCOVERY Purple detection. Finally, nuclei were subsequently visualized with hematoxylin. The images were taken with Aperio ImageScope. We semi-quantitively scored the slides based on the intensity of the brown color (HuR): weak, moderate, and strong. The intensity of the HuR was also analyzed by the Aperio Positive Pixel Count Algorithm v9.

### 2.5. Cell Culture

Primary human lung fibroblasts (HLFs) were isolated from lung tissue by explant procedure [33]. Cells utilized in this study were derived from non-smokers (control; M/F = 1/3; age 71 ± 7 years), current smokers with COPD (M/F = 2/2; age 61 ± 5.4 years) and without COPD (smoker; M/F = 1/3; age 61.3 ± 4.8 years). Experiments were conducted with fibroblasts from 3–4 different individuals of each patient group unless otherwise indicated and were between passages five to eight. All fibroblast strains were cultured and analyzed at the same time and were within one passage to assess the basal expression levels of HuR.

### 2.6. Western Blot

HLFs were grown to approximately 70–80% confluence and cultured with serum-free MEM for 18 h before the treatment. Total cellular protein was extracted using RIPA lysis buffer (ThermoFisher Scientific, Waltham, MA, USA) and Protease Inhibitor Cocktail (PIC, Roche, Laval, QC, Canada). Nuclear and cytoplasmic fractions were extracted using a nuclear extract kit (Active Motif, Carlsbad, CA, USA). Ten to twenty μg (for ACE2 and COX-2 detection) or 60 μg (for cleaved HuR detection) of protein lysate were subjected to 10% SDS-PAGE gels and transferred onto Immuno-blot PVDF membranes (Bio-Rad Laboratories, Mississauga, ON, Canada), as previously described [34]. Then, the membrane was blocked for one hour at room temperature in blocking solution (5% *w*/*v* of non-fat dry milk in 1× PBS/0.1% Tween-20). Antibodies against HuR (1:2000; Santa Cruz, Dallas, TX, USA), ACE2 (SN0754; 1:500–1:1000; Invitrogen, Waltham, MA, USA), COX-2 (1:1000; Cell Signaling Technologies, Whitby, ON, Canada), Laminin A/C (1:1000; Cell Signaling Technologies, Whitby, ON, Canada) and β-Tubulin (1:50,000; Sigma, St. Louis, MO, USA) were used. Secondary antibodies goat anti-rabbit IgG HRP-linked (1:10,000, Cell Signaling Technologies) and HRP-conjugated horse anti-mouse IgG (1:10,000, Cell Signaling Technologies, Whitby, ON, Canada) were used. Detection of protein was catalyzed by enhanced chemiluminescence (ECL) and visualized using a ChemiDoc™ MP Imaging System (Bio-Rad Laboratories, Mississauga, ON, Canada). Densitometric analysis was performed using Image Lab™ Software Version 5 (Bio-Rad, Mississauga, ON, Canada). Protein expression was normalized to β-Tubulin and the data presented as the fold-change relative to the untreated condition.

### 2.7. Quantitative RT-PCR (RT-qPCR)

Total RNA was isolated using miRNeasy Kits (QIAzol based RNA purification, Qiagen) according to the manufacturer’s instructions. Quantification was conducted on a Nanodrop 1000 spectrophotometer (infinite M200 pro, TECAN, San Jose, CA, USA). Reverse transcription of RNA was carried out using iScript™ Reverse Transcription Supermix (Bio-Rad Laboratories, Mississauga, ON, Canada). Then, mRNA levels of *ELAVL1*, *ACE2* and *S9* were analyzed using this cDNA template and gene-specific primers (Table 1). Quantitative PCR (qPCR) was performed by addition of 1 µL cDNA and 0.5 µM primers with SsoFast™ EvaGreen^®^ (Bio-Rad Laboratories, Mississauga, ON, Canada), and PCR amplification was performed using a CFX96 Real-Time PCR Detection System (Bio-Rad Laboratories, Mississauga, ON, Canada). Thermal cycling was initiated at 95 °C for 3 min and followed by 39 cycles denaturation at 95 °C for 10 s and annealing at 59 °C for 5 s. Gene expression was analyzed using the ΔΔC_T_ method, and results are presented as fold-change normalized to the housekeeping gene (*S9*).

### 2.8. RNA Immunoprecipitation-qPCR (RIP-qPCR)

HLFs were grown to approximately 70–80% confluence and cultured with serum-free MEM for 18 h before collection. Cells were collected in PBS and centrifuged at 1500 rpm at 4 °C for 5 min. The cell pellets were lysed (50 mM Tris PH 8; 0.5% Triton X100; 450 mM NaCl; Protease Inhibitor Cocktail; Phosphatase Inhibitor (Roche, Laval, QC, Canada), incubated for 15 min on ice, and then centrifuged at 10,000 rpm, 4 °C for 15 min. The extracts were transferred into as new tube and a buffer containing 50 mM Tris pH 8; 0.5% Triton X100; 10% glycerol; Protease Inhibitor Cocktail; Phosphatase Inhibitor (Roche, Laval, QC, Canada) [35,36] was added. Protein concentration was measured by the BCA Protein Assay Kit. Thirty-five μL of protein G Sepharose^TM^ 4 fast glow beads (GE Healthcare, Mississauga, ON, Canada) were pre-coated with 3 μg of IgG (Cell Signaling Technologies, Whitby, ON, Canada) or 3 μg of anti-HuR (Santa Cruz Biotechnology, Dallas, TX, USA) antibodies overnight on a rotator at 4 °C. Beads were washed three times with buffer (50 mM Tris PH 8; 0.5% Triton X100; 150 mM NaCl) and incubated with cell extracts for 2 h a 4 °C. Beads were washed three times to wash out unbound materials. RNA was then extracted, reverse transcribed and analyzed by qPCR (RT-qPCR), as described above. RNA expression was normalized to *S9* mRNA bound in a non-specific manner to IgG.

### 2.9. HuR-esiRNA Knockdown in Human Lung Fibroblasts

HLFs were seeded at 10 × 10^4^ cells/cm^2^ and transfected with 60 pmol of endoribonuclease prepared siRNA (esiRNA) against *ELAVL1* (MISSION^®^ esiRNA, Sigma, St. Louis, MO, USA) or non-targeting control esiRNA (MISSION^®^ esiRNA, Sigma) with Lipofectamine RNAiMAX transfection reagent (ThermoFisher Scientific, Waltham, MA, USA) in accordance with the manufacturer’s instructions. One hour after the transfection, 10% MEM medium was added on the cells. After 24 h, the cells were switched to serum-free MEM for 44 h, after which cellular proteins were collected. Confirmation of HuR knockdown was done by Western blot within 68 h after transfection.

### 2.10. Determination of mRNA Stability

HLFs were serum-starved for 18 h followed by treatment of Actinomycin D (ActD-1 µg/mL) (Enzo Life Sciences, Toronto, ON, Canada), an inhibitor of RNA synthesis [28,37], for 0, 3, 6, and 9 h, after which RNA was collected; qPCR was performed as described above to determine the remaining levels of *ACE2* mRNA. The concentration of ActD used in this experiment did not affect cell viability (data not shown) but blocked transcription [38].

### 2.11. Determination of Protein Stability

HLFs were serum-starved for 18 h followed by the protein collection for the 0-h time point. The remaining cells were treated with cycloheximide (CHX-1 µg/mL)—an inhibitor of protein synthesis [39]—for 4, 8, 24, and 48 h. Total protein was harvested, and Western blot was performed to determine the remaining levels of ACE2 protein. CHX concentration used in this experiment did not affect cell viability (data not shown).

### 2.12. Preparation of Cigarette Smoke Extract (CSE)

Research grade cigarettes (3R4F) with a filter were acquired from the Kentucky Tobacco Research Council (Lexington, KT, USA). Each cigarette contains 0.73 mg of nicotine, 9.4 mg of tar, and 12.0 mg of CO, as described by the manufacturer. CSE was produced as previously described [28,32,38,40,41].

### 2.13. Imaging Flow Cytometry

HLFs were grown to approximately 70–80% confluence and cultured with serum-free MEM for 18 h before the treatment. Cells were exposed to 2% CSE for 4 h. Then, cells were trypsinized, collected, and prepared according to the manufacturer’s instructions. Briefly, after washing cells with PBS-0.2% BSA, cells were fixed with 4% paraformaldehyde (PFA) for 20 min at room temperature. After incubation, PBS-0.2% BSA was added, spun down at 400× *g* at 4 °C for 5 min, and the supernatant was discarded. The pellet was resuspended in 400 μL PBS-0.2% BSA, spun down at 400× *g* at 4 °C for 5 min, and again the supernatant was discarded. Then, the pellet was resuspended in 100 μL of permeabilization buffer (BD Perm/Wash^TM^ Buffer, eBioscience™), and was incubated for 15 min at room temperature. After incubation, 1 μL of PE mouse anti-HuR (1:100, BD Pharmingen™) was added and incubated for 30 min at 4 °C in the dark, and then 1 mL of permeabilization buffer was added. The samples were centrifuged, and the pellet was resuspended in 75 μL of 1× PBS-0.2% BSA. The samples were filtered and kept in the dark. Before acquiring the samples, 5 μL Hoechst (1: 20,000, Hoechst 3342, ThermoFisher Scientific, Waltham, MA, USA) was added. In each experiment 20,000 events for each sample were acquired using a 12 channel Amnis^®^ ImageStream^®X^ Mark II (EMD Millipore, Oakville, ON, Canada) imaging flow cytometer equipped with the 405 nm and 488 nm lasers. Samples were gated to remove debris, and 20,000 event/sample were analyzed using IDEAS^®^. After gating the cytoplasmic fraction, intensity was used to evaluate HuR expression.

### 2.14. Statistical Analysis

Using GraphPad Prism 6 (v. 6.02; La Jolla, CA, USA), statistical analysis was performed using a non-parametric one-way analysis of variance (ANOVA) followed by Dunn’s multiple comparisons test to assess the differences between the non-smoker, smoker, and COPD subjects as well as between treatments of more than two. Groups of two were analyzed by the one- or two-tailed unpaired t-test, as described below. A two-way analysis of variance (ANOVA), followed by Sidak’s multiple comparisons test was used to evaluate differences between groups and conditions of more than two. Results are presented as means ±standard error of the means (SEM) of the fold-changes relative to control cells. In all cases, *p* values < 0.05 were considered statistically significant. Mann–Whitney U tests were also used in single-cell RNA-seq to quantify the difference in *ELAVL1* gene expression between different cell populations.

## 3. Results

### 3.1. Expression of ELAVL1 and ACE2 in Cells of the Upper and Lower Respiratory System

**Figure 1:**
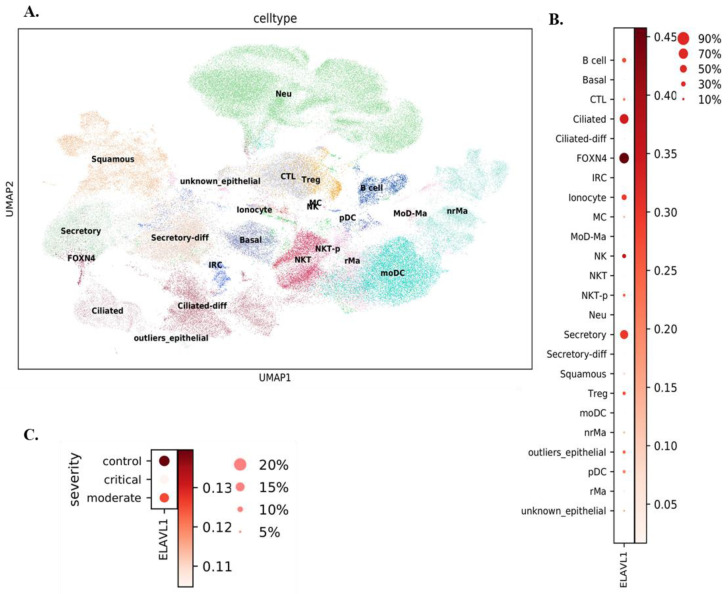
*ELAVL1* single-cell level expression in nasopharyngeal and bronchial cells. (**A**) Different cell populations in an existing single-cell RNA-seq data [30] were analyzed as described in the Materials and Methods. (**B**) Dotplot representing the expression of *ELAVL1* in different cell populations. The size of the circle denotes the % of cells expressing the *ELAVL1* gene. The darkness of the circle shows the expression level. The expression of *ELAVL1* is significantly higher (Mann–Whitney U test, *p*-value = 0) in ciliated, FOXN4, secretory cells than that in any other cells. (**C**) The expression of *ELAVL1* is significantly lower (Mann–Whitney U test, *p*-value = 4.41 × 10^−23^) in critical COVID-19 patients than in moderate and control patients.

Comprehensive expression analysis of *ELAVL1* in pulmonary cell populations has not been done. Therefore, we first analyzed existing single-cell RNA-seq datasets to comprehensively profile *ELAVL1* in cells along the respiratory tract. The first dataset utilized nasopharyngeal/bronchial cells from 19 SARS-CoV-2-positive patients and five SARS-CoV-2-negative donors [30]. Conducting airway epithelial cells, (basal, secretory, ciliated, FOXN4+ cells, and ionocytes) and immune cells (macrophages, dendritic cells, and natural killer (NK) cells) were among the cells detected (Figure 1A). Comparing *ELAVL1* expression in these different cell populations in COVID-19 patients revealed that the expression of *ELAVL1* is significantly higher (Mann–Whitney U test, *p*-value = 0) in ciliated, FOXN4+, and secretory cells (Figure 1B) and that there was differential *ELAVL1* expression based on COVID-19 severity (Figure 1C; Mann–Whitney U test, *p*-value = 4.41 × 10^−23^.

**Figure 2:**
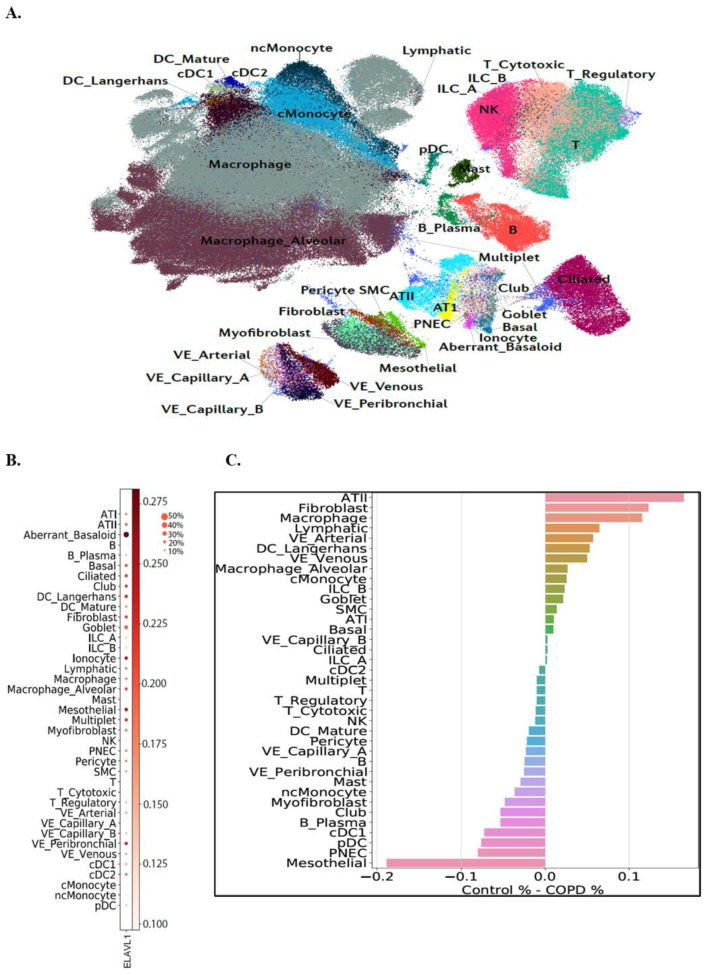
*ELAVL1* single-cell level expression in COPD. (**A**) Different cell populations in the COPD single-cell RNA-seq data [29]. (**B**) Dotplot shows the expression of *ELAVL1* in different cell populations. The size of the circle denotes the % of cells expressing *ELAVL1*. The darkness of the circle shows the expression level. Aberrant_basaloid, mesothelial, VE_peribronchial, PNEC, ciliated, and club cells are among the cell types with the highest expression of *ELAVL1*. (**C**) Percentage of *ELAVL1*+ cells: on the right side (positive % difference) is the cell types with higher % of *ELAVL1*+ cells in control. On the left side are the cell types with higher % of *ELAVL1*+ cells in COPD (0.1 denotes 10%).

It is also unknown whether *ELAVL1* expression changes with COPD. Therefore, we also assessed *ELAVL1* expression in lung cells from COPD patients using existing single-cell RNA-seq expression data on samples obtained from 28 control donor lungs and 18 COPD lungs [29]. The cell populations identified are in Figure 2A. In COPD, *ELAVL1* expression is higher in aberrant basaloid, mesothelial, peribronchial vascular endothelial (VE), pulmonary neuroendocrine cell (PNEC), ciliated, and club cells, relative to other cell types (Figure 2B). Further, we found that mesothelial, PNEC, and myofibroblasts have relatively high *ELAVL1* expression in COPD, whereas alveolar type II (ATII), fibroblasts, and macrophages are among the cell types with relatively high % of *ELAVL1*+ cells in control (Figure 2C).

**Figure 3:**
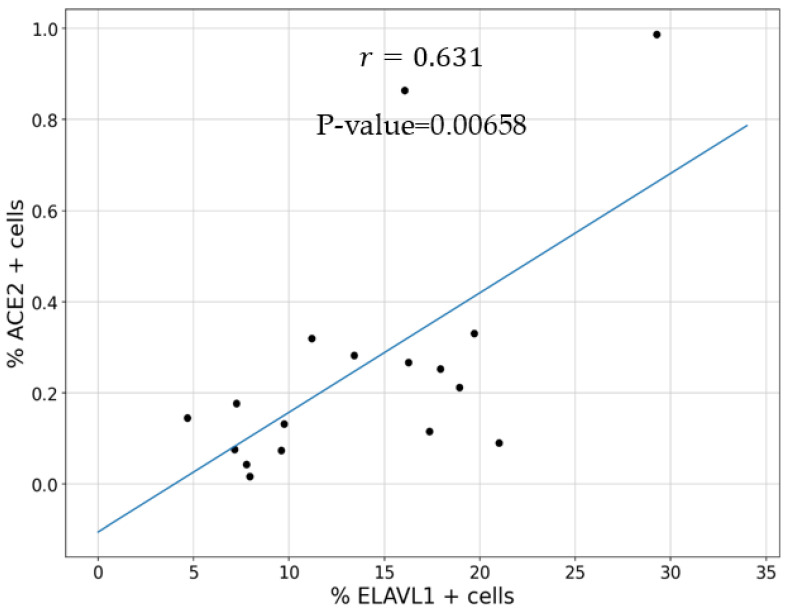
Correlation between *ELAVL1* and *ACE2* in COPD. The percentage of *ELAVL1* and *ACE2* positive cells in all cell types of the COPD samples were analyzed. There was a significant (*p*-value < 0.01) correlation (Pearson correlation coefficient = 0.631) between *ELAVL1* and *ACE2*. Only cell types expressing both *ELAVL1* and *ACE2* were included in the analysis.

Finally, we explored whether there was a correlation between *ELAVL1* and *ACE2* expressions in COPD cells that express both genes. We found a significant positive correlation (Pearson correlation coefficient = 0.631; *p*-value < 0.01) between *ELAVL1* and *ACE2* expression (Figure 3). In summary, *ELAVL1* expression varies amongst various pulmonary cell types and disease phenotypes but there is a positive correlation between *ELAVL1* and *ACE2* expression in COPD.

### 3.2. Cytoplasmic Localization of HuR Is Increased in Smoker and COPD Lung Cells

**Figure 4:**
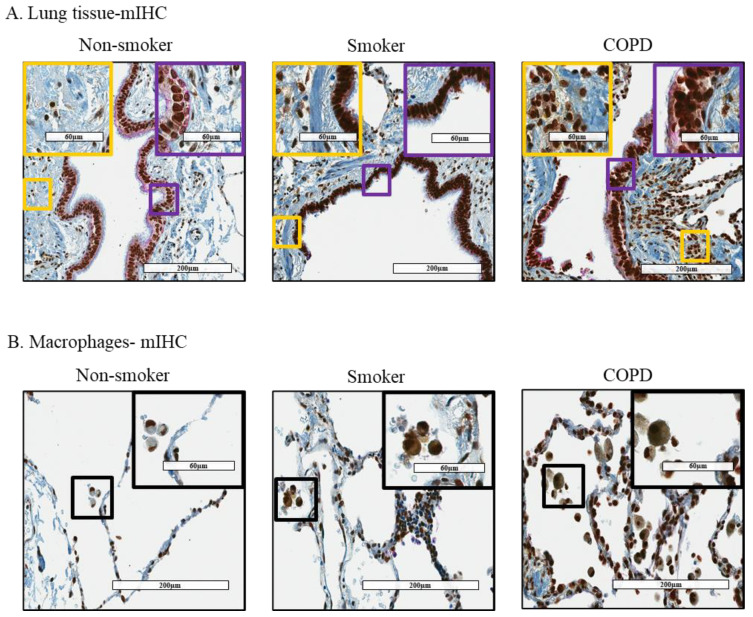
Cytoplasmic expression of HuR increased in lung tissue and macrophages from smoker and COPD subjects. (**A**) Lung tissue—mIHC: lung tissue was stained with mIHC: HuR was stained with brown color, Cytokeratin 19 expression (a marker of epithelial cells) was stained with purple color, and vimentin (a marker for fibroblasts) was stained yellow. There was more HuR in the cytoplasm of epithelial cells and fibroblasts from smoker and COPD subjects comparing to these cells from non-smoker individuals. (**B**) Macrophages—mIHC: there was an increase in cytoplasmic HuR in macrophages from smoker and COPD subjects. The pictures were taken by Aperio ImageScope with 20×, and 40× for higher magnification. Images are representative for 3 to 4 subjects/group.

We next used mIHC to detect HuR (brown) in epithelial cells (purple) and fibroblasts (yellow) (Figure 4A,B). In lungs of non-smokers, HuR was localized predominantly in the nucleus of epithelial cells and fibroblasts while only weak cytoplasmic HuR expression was detected in both cell types (Figure 4A–left panel). Prominent HuR expression was detected in lung cells of smokers with and without COPD (Figure 4A–middle and right panels). We also found that cytoplasmic HuR expression was relatively high in pulmonary macrophages of smokers and COPD subjects (Figure 4B). These data indicate differential localization of HuR in the lungs in response to cigarette smoke, with there being prominent cytoplasmic levels.

### 3.3. Protein Expression of HuR Is Similar between Non-Smoker, Smoker, and COPD-Derived Lung Fibroblasts

**Figure 5:**
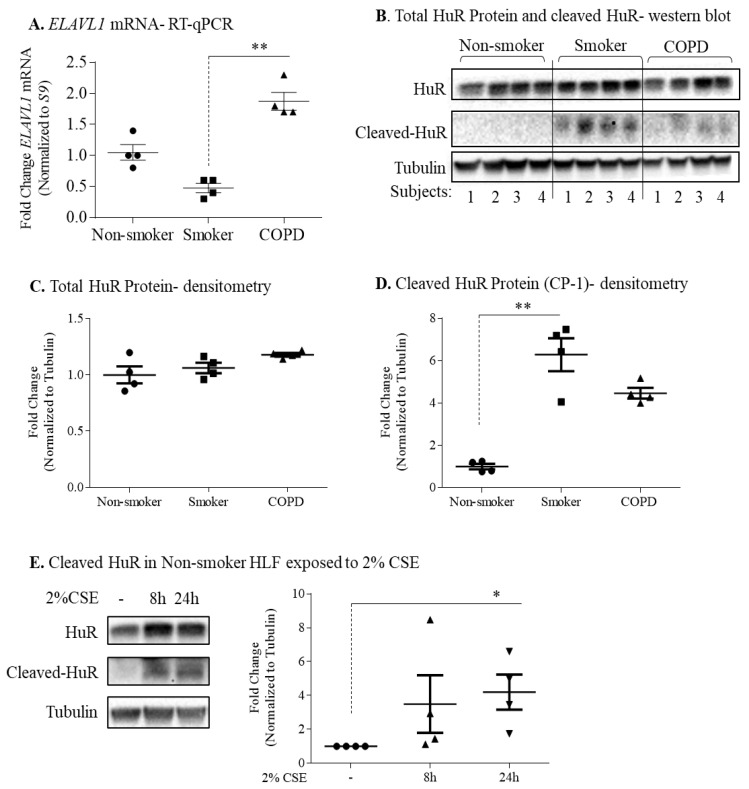
HuR expression in non-smoker, smoker, and COPD HLFs. (**A**) *ELAVL1* mRNA-RT-qPCR: HLFs from 4 non-smoker (circle), 4 smoker (square), and 4 COPD (triangle) subjects were harvested for RT-qPCR. There was a significant difference between smoker and COPD-derived HLFs (** *p* < 0.01). Results are expressed as the mean ± SEM. (**B**) Total HuR Protein—Western blot- densitometry: HLFs from 4 non-smoker, 4 smoker, and 4 COPD subjects were harvested for Western blot. HuR protein expression was detected at the predicted MW of 34 kDa. The cleaved HuR product (CP-1, 27 kDa) was detected in HLFs from smoker and COPD subjects. β-Tubulin was used as loading control. (**C**) Total HuR Protein—densitometry: there was no significant difference in total expression of HuR between non-smoker (circle), smoker (square), and COPD (triangle) HLFs. Results are expressed as the mean ± SEM. (**D**) Cleaved HuR Protein (CP-1)—densitometry: there was significant increase in the cleaved HuR (CP-1) in smoker (square) HLFs comparing to non-smoker (circle) HLFs (** *p* = 0.009). Results are expressed as the mean ± SEM. (**E**). Cleaved HuR in non-smoker HLFs exposed to 2% CSE: there was an increase in the cleaved HuR product (CP-1) in HLFs from non-smokers exposed to 2% CSE for 8 h and 24 h (* *p* = 0.01). Results are expressed as the mean ± SEM of 4 independent experiments.

We used HLFs to mechanistically evaluate the involvement of HuR in controlling ACE2 expression, as we have previously shown that HuR protein is constitutively expressed in these cells [28] and that there is more ACE2 protein in COPD-derived lung fibroblasts [9]. *ELAVL1* mRNA levels were significantly higher in COPD-derived HLFs relative to the smoker-derived cells (Figure 5A). In the non-smoker, smoker, and COPD-derived HLFs, HuR protein was detected at its predicted molecular weight (MW) of ~34 kDa (Figure 5B). There was no significant difference in HuR protein expression between the three groups (Figure 5C). Interestingly, another ~27 kDa protein band was detected only in the smoker and COPD-derived cells (Figure 5B) that likely reflects cleaved HuR (CP-1) [42]; CP-1 expression is significantly higher in smoker comparing to non-smoker HLFs (Figure 5D). Then, we evaluated the effect of cigarette smoke extract (CSE) on the expression of HuR in non-smoker-derived HLFs by treating cells with 2% CSE for 6 h, 8 h, and 24 h. There was no difference in total HuR protein expression in response to 2% CSE (Figure 5E). However, there was a significant increase in cleaved HuR (CP-1) in response to 2% CSE for 24 h in non-smoker-derived HLFs (Figure 5E). Together, these data indicate that cigarette smoke does not change the total HuR protein in HLF but induces its cleavage.

### 3.4. Increased ACE2 Protein in COPD-Derived Lung Fibroblasts Is Not Associated with Changes at the mRNA Level or Increased Binding to HuR

**Figure 6:**
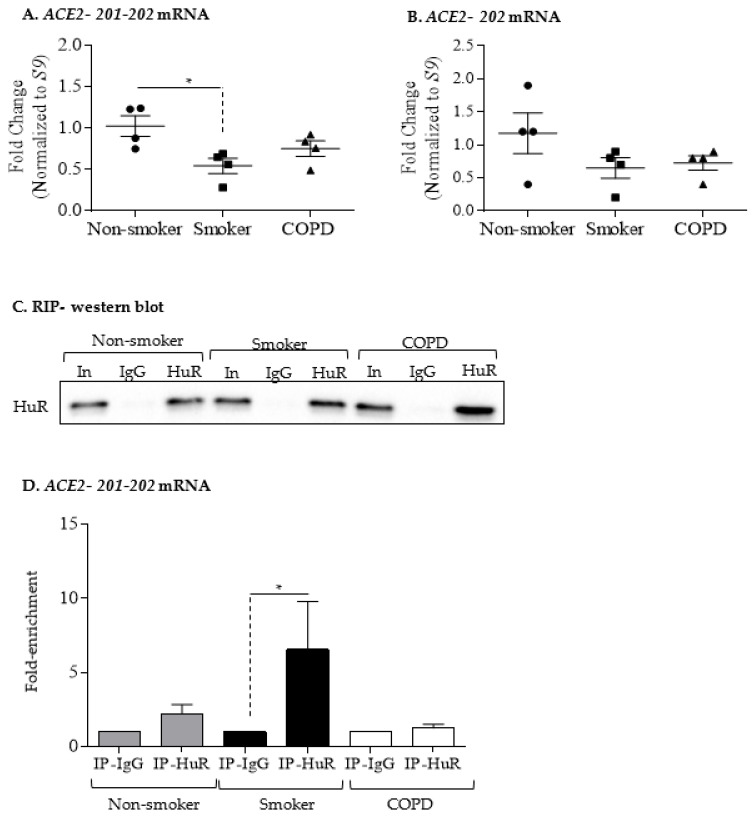
*ACE2* mRNA expression and binding to HuR. (**A**) *ACE2-201-202* mRNA: there was significantly less mRNA in smoker (triangle) compared to non-smoker (circle) HLFs (* *p* < 0.05) (**B**) *ACE2-202* mRNA: there was no significant difference between the three groups of HLFs. (**C**) RIP—Western blot: representative Western blot of HuR IP is shown. Input (In) refers to cell lysates. IP-IgG refers to immunoprecipitation (IP) with control IgG antibody while IP-HuR refers to the IP with anti-HuR IgG antibody. Note the presence of HuR protein in IP-HuR but not in IP-IgG. (**D**) *ACE2-201-202* mRNA: detection of *ACE2-201-202* mRNA in IP-IgG and IP-HuR was done using qPCR. Values are expressed as fold enrichment to values measured in IP-IgG. The enrichment of *ACE2-201-202* in IP-HuR of HLFs from non-smoker and smoker but not in COPD. Results are presented as the mean ± SEM) (* *p* < 0.05 compared to IP-IgG).

We next examined if there was differential expression of ACE2 at the mRNA level. Using two different primers sets for human *ACE2* (*ACE2*-*201*-*202* and *ACE2*-*202*), we found that there was a significant difference in *ACE2* mRNA levels between quiescent HLFs derived from non-smoker and smoker lungs for *ACE2*-*201*-*202* (Figure 6A) but not for *ACE2*-*202* (Figure 6B). Overall, this suggests that changes at the transcriptome level are unlikely to account for higher ACE2 protein in COPD-derived HLFs.

HuR binds to target mRNA, thereby increasing stability of the transcript [25]. It is not known whether HuR associates with *ACE2* mRNA. To address this, we assessed HuR association with *ACE2* mRNA by immunoprecipitation of HuR followed by RT-qPCR analysis of *ACE2* mRNA. Western blot analysis showed that HuR immunoprecipitated from HLF cell extracts similarly between non-smoker, smoker, and COPD (Figure 6C). Note the specificity of the IP, as the immunoglobulin G (IgG) antibody did not immunoprecipitate HuR, as indicated by the absence of a band on the Western blot. Furthermore, HuR strongly bound *ACE2-201-202* mRNA in smoker-derived HLFs (Figure 6D). HuR did not associated with *ACE2-202* mRNA (data not shown). Thus, HuR can bind *ACE2* mRNA in HLFs.

### 3.5. HuR Does Not Control ACE2 mRNA or Protein Stability

**Figure 7:**
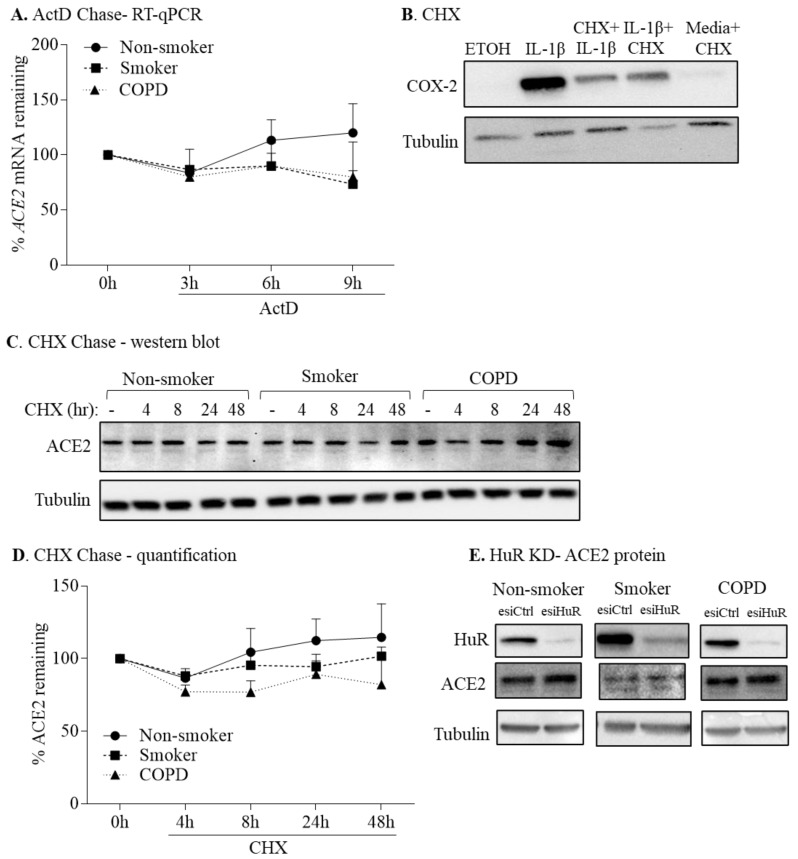
*ACE2* mRNA and protein stability are not controlled by HuR. (**A**) ActD Chase—RT-qPCR: HLFs from 3 non-smoker, 3 smoker, and 3 COPD subjects were exposed to ActD (1 µg/mL) for the indicated time point. *ACE2* levels were set to equal 100% after starvation for 18 h (0 h) and are expressed as percentage (%) of *ACE2* mRNA remaining. In non-smoker, smoker, and COPD HLFs, *ACE2* mRNA was similar after exposure to ActD for 3 h, 6 h, and 9 h compared to time 0. (**B**) CHX: HLFs were exposed to ethanol (control), IL-1β (1 ng/mL) alone for 24 h, pretreated with CHX (1 µg/mL) for 1 h followed by IL-1β for 24 h, or pretreated with IL-1β for 24 h followed by CHX for 24 h. COX-2 protein expression was reduced by CHX. Representative Western blot is shown. (**C**) CHX Chase—Western blot: HLFs from 3 non-smoker, 3 smoker, and 3 COPD subjects were exposed to CHX (1 µg/mL) for the indicated time point. Note that there is no change in ACE2 upon CHX treatment. (**D**) CHX chase—quantification: ACE2 levels were set to equal 100% after starvation for 18 h (0 h) and are expressed as percentage (%) of ACE2 remaining. In non-smoker, smoker, and COPD HLFs, ACE2 protein was similar after exposure to CHX for 4 h, 8 h, 24 h, and 48 h compared to time 0. Results are expressed as the mean ± SEM. (**E**) HuR KD-ACE2 protein: HuR knockdown had minimal effect on basal ACE2 protein levels in lung fibroblasts. Representative Western blots are shown.

To evaluate stability of the *ACE2* transcript between non-smoker, smoker, and COPD-derived HLFs, we treated these cells with actinomycin D (ActD 1 µg/mL), an inhibitor of RNA synthesis [28,37], for 3, 6, and 9 h, followed by an analysis of *ACE2* mRNA. There was no significant difference in the rate of *ACE2* mRNA decay between cells from the three subject groups (Figure 7A). There was also no change in the rate of *ACE2* mRNA decay within cells from subjects within a group. This indicates that *ACE2* mRNA expression is unlikely to be controlled at the level of mRNA stability in HLFs.

To evaluate ACE2 protein stability, we exposed HLFs from non-smoker, smoker, and COPD subjects to cycloheximide (CHX 1 µg/mL), an inhibitor of protein synthesis [39], for up to 48 h, and assessed ACE2 protein expression by Western blot. To ensure that protein synthesis was indeed blocked by CHX, we include data demonstrating that both pre-and post-treatment with CHX attenuated IL-1β-induced COX-2 expression (Figure 7B). CHX did not significantly change ACE2 protein levels in HLFs. In addition, no significant difference in ACE2 protein stability between non-smoker, smoker, and COPD-derived lung fibroblasts were observed (Figure 7C,D). These results indicate that increased ACE2 expression in COPD-derived HLFs is not due to alterations in mRNA or protein stability.

To confirm HuR involvement in basal ACE2 expression, we transiently transfected HLFs derived from non-smoker, smoker, and COPD with control esiRNA (esiCtrl) or HuR-specific esiRNA (esiHuR). Basal HuR expression declined by ~80% in cells transfected with esiHuR-relative to esiCtrl-transfected cells (Figure 7E) but there was no significant difference in ACE2 protein levels in esiHuR-transfected cells relative to those transfected with esiCtrl in the three groups, suggesting that HuR does not play a dominant role in regulating ACE2 protein expression in quiescent HLFs (Figure 7E).

### 3.6. Non-Smoker and COPD-Derived Lung Fibroblasts Exposed to CSE Exhibit Increased Cytoplasmic HuR

**Figure 8:**
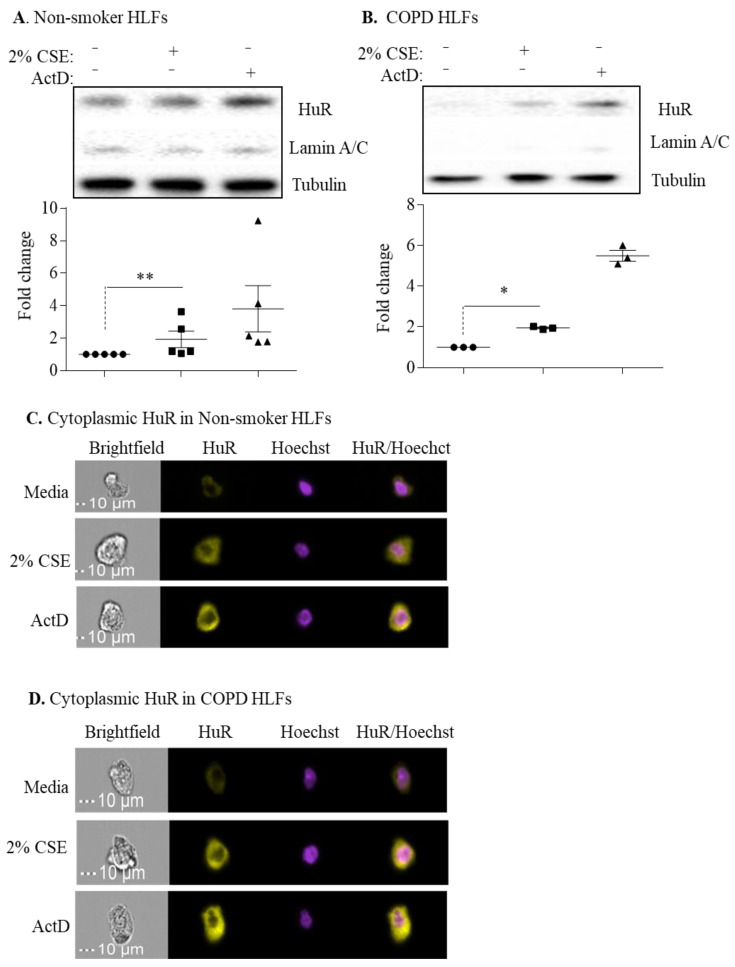
Cytoplasmic HuR is increased by cigarette smoke. (**A**) Cytoplasmic HuR in non-smoker HLFs: there was an increase in HuR cytoplasmic localization in response to 2% CSE for 4 h (** *p* = 0.004). Actinomycin D (ActD) was used as positive control for the translocation of HuR. Lamin A/C is a nuclear marker, while tubulin is a cytoplasmic marker. Results are expressed as the mean ± SEM of 5 independent experiments. (**B**) COPD HLFs: there was an increase in HuR cytoplasmic localization in response to 2% CSE for 4 h (* *p* < 0.05). Results are expressed as the mean ± SEM of 3 independent experiments. Data between untreated and CSE-exposed cells were analyzed by a Mann–Whitney one-tailed *t*-test. (**C**) Cytoplasmic HuR in non-smoker HLFs: HuR localization in non-smoker HLFs treated with 2% CSE was assessed by Imaging Flow Cytometry. There was an increase in HuR expression in response to 2% CSE for 4 h. ActD was used as a positive control for HuR translocation into the cytoplasm. A representative picture for cells is shown from 2 independent experiments. (**D**) Cytoplasmic HuR in COPD HLFs: there was an increase in HuR expression in the cytoplasm of COPD HLFs exposed to 2% CSE for 4 h. A representative picture for cells is shown from one COPD subject.

**Figure 9:**
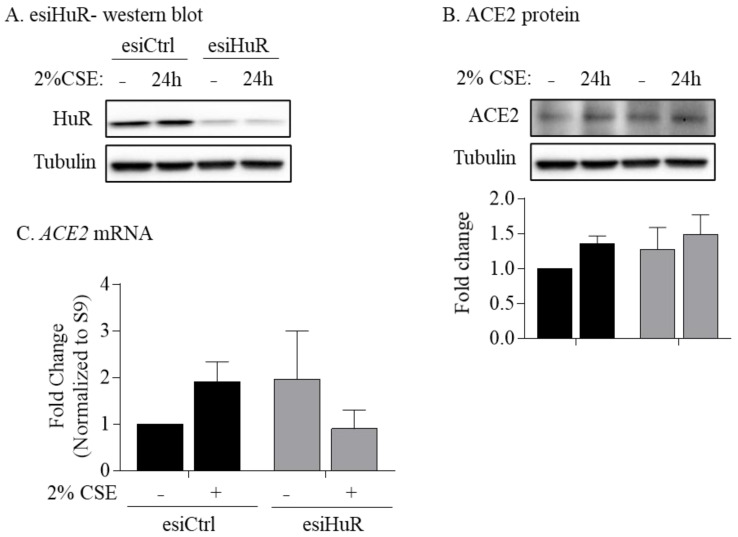
HuR silencing does not affect protein and mRNA expression of ACE2. (**A**) HuR protein—Western blot: transfection of non-smoker HLFs with esiHuR reduced the level of HuR protein by ~80%. (**B**) ACE2 protein: there was no significant difference in ACE2 protein levels in esiHuR-transfected HLFs exposed to 2% CSE for 24 h compared to untreated esiHuR-transfected cells and to esiCtrl-transfected cells. Results are expressed as the mean ± SEM of 3 independent experiments (HLFs used from one non-smoker subject). (**C**) *ACE2* mRNA: there was no significant difference in *ACE2* mRNA in esiHuR-transfected HLFs exposed to 2% CSE for 24 h compared to untreated esiHuR-transfected cells and to esiCtrl-transfected cells. Results are expressed as the mean ± SEM of 3 independent experiments (HLFs used from one non-smoker subject).

Finally, to directly assess whether HuR regulates ACE2 expression in response to cigarette smoking, we used CSE, an in vitro surrogate of cigarette smoke exposure. Cytoplasmic HuR protein levels detected by Western blotting significantly increased in response to acute 2% CSE exposure (Figure 8A,B); ActD was used as a positive control. To confirm the effect of CSE on HuR localization, we used imaging flow cytometry (ImageStream^®^). There was a noticeable increase in cytoplasmic HuR levels in response to 2% CSE (4 h) in HLFs derived from non-smoker (Figure 8C) and COPD (Figure 8D), suggesting that acute cigarette smoke exposure induces translocation of HuR to the cytoplasm of primary HLFs. Finally, we conducted knockdown experiments to evaluate if HuR regulates ACE2 expression in response to CSE. Verification of HuR knockdown is shown in Figure 9A. HuR knockdown had no effect on ACE2 protein or mRNA levels in the absence and presence of CSE exposure (Figure 9B,C). Thus, we show the differential expression of *ELVAL1* (HuR) in lung cell populations and show that the regulation of ACE2 is unlikely to be mediated by HuR despite activation of HuR by cigarette smoke. Further insight into the molecular regulation of ACE2 is needed to facilitate the development of new medications to combat coronavirus infections.

## 4. Discussion

COPD is a major health problem with limited therapeutic options [1] that is primarily caused by cigarette smoke exposure [4]. COPD patients may be at risk of increased hospitalization and severe illness from COVID-19 caused by SARS-CoV-2 [5]. One possible reason behind increased susceptibility may be the upregulation of ACE2, the entry receptor for SARS-CoV-2 [6,7]. However, the mechanism(s) involved in the upregulation of ACE2 are poorly understood. Therefore, in this study, we investigated the role of HuR in the regulation of pulmonary ACE2. We also sought to comprehensively profile the expression of HuR/*ELAVL1* in the various cells of lungs from COPD and COVID-19 patients. Further, we predicted HuR would regulate expression of the ACE2 protein. Herein, we describe the differential regulation of *ELAVL1* in distinct populations of pulmonary cells and the extensive cytoplasmic localization of HuR in COPD lungs. We found that there was a significant positive correlation between *ELAVL1* and *ACE2* in COPD. While we speculate that the increased cytoplasmic HuR in COPD lungs drives features of disease pathology (e.g., inflammation), our data show that HuR is not likely to be involved in the regulation of ACE2 protein expression.

Our rationale for focusing on HuR is the fact that RBPs regulate mRNA post-transcriptional events of genes involved in various cellular mechanisms [43,44], including viral infection [45]. A recent study showed that SARS-CoV-2 RNA binds to 104 human proteins, including ribosomal proteins, translation factors, and RBPs [46]. Cellular nucleic acid-binding protein (CNBP) is one of the direct interactors with SARS-CoV-2 RNA. Another RBP that binds to SARS-CoV-2 RNA is la-related protein 1 (LARP1). In a LARP1 knockout cell line, intracellular viral RNA and the infectious virus production are elevated. In contrast, LARP1 overexpression decreases viral RNA and the levels of infectious virus [46]. Furthermore, the alphavirus Sindbis virus, a single-stranded positive-sense RNA virus that contains multiple U-rich elements, recruits cellular HuR to stabilize its viral RNA to aid in the expression viral proteins and maintain infection. Sindbis virus infection also induces HuR cytoplasmic translocation where the viral RNAs accumulate [47]. HuR also associates with the U-rich elements of hepatitis C virus (HCV) RNA [48] and the late regulatory element (LRE) of human papillomaviruse 16 (HPV16), with HuR depletion reducing L1 capsid protein expression [49]. Because Sindbis virus has high affinity to HuR, it sequestrates HuR in the cytoplasm. Consequently, the alternative pre-mRNA polyadenylation and splicing of cellular pre-mRNAs as well as the stability of a subset of cellular mRNAs are dysregulated during Sindbis virus infection [50]. These studies provide rationale for investigating the function of RBPs in the context of viral infection, and they may be crucial to our understanding of infectivity, particularly in light of the ongoing COVID-19 pandemic.

We found that cytoplasmic localization of HuR is increased in lung structural cells, mainly epithelial cells and fibroblasts, in lung tissue from smoker and COPD subjects. These findings recapitulate those of a previous study showing cytoplasmic HuR is elevated in lung tissue from smoker and COPD patients [51] and suggests that smoking itself influences the cellular localization of HuR in the lung. We also found that macrophages from smoker and COPD subjects have higher cytoplasmic localization of HuR compared to macrophages from non-smokers. Macrophages are involved in the pathophysiology of COPD via the production of inflammatory mediators and proteases [52]. Alveolar macrophages are also involved in the pathogenesis of SARS-CoV-2 [53]. Matrix metalloproteinase-9 (MMP-9) is increased in COPD and by SARS-CoV-2 infection that may be involved in alveolar destruction [54,55]. HuR stabilizes *MMP-9* mRNA and induces its protein expression [56]. Increased cytoplasmic expression of HuR also stabilizes tumor necrosis factor-α (*TNF*-*α*) and interlukin-6 (*Il*-*6*) mRNA in macrophages [57], two pro-inflammatory mediators that are also increased in COPD and SARS-CoV-2 infection [54,58]. HuR may also be involved in the pathogenesis of COVID-19 by its ability to increase inflammation, as SARS-CoV-2 infection induces TNF-α, IL-6, and CC-chemokine ligand 2 (CCL2) [58,59].

Although *ELAVL1* mRNA is significantly increased in COPD compared to smoker, the protein levels are similar between the two groups. This decoupling of protein and mRNA expression in COPD suggests that elevated mRNA levels in COPD might because of the increase in *ELAVL1* transcription and/or its mRNA stability. However, the regulation of *ELAVL1* at transcriptional and/or at post-transcriptional levels in smoker and COPD is not known yet. Other possible mechanism by which *ELAVL1* mRNA levels may rise in the absence of their translation is the sequestration of these transcripts in stress granules (SGs), which are cytoplasmic ribonucleoprotein complexes that assemble in response to stress and sequester mRNAs [60]. Currently, we do not know about SGs assembly during cigarette smoke exposure and in COPD.

Our notion that HuR could also control ACE2 expression was based in part on our recent report demonstrating increased ACE2 expression in COPD-derived lung fibroblasts, and that pulmonary ACE2 protein increases in response to chronic cigarette smoke exposure [9]. Lung fibroblasts provide structure and support to the lungs by synthesizing and maintaining an extracellular matrix (ECM) [61], and in the context of chronic inflammation, activation of fibroblasts leads to the production of several cytokines and chemokines [62]. We found that CSE induced HuR translocation to the cytoplasm and that HuR binds to *ACE2* mRNA in lung fibroblasts from smoker, but silencing HuR had no effect on *ACE2* mRNA and protein. This is consistent with a previous observation that HuR binds to *PTGS2* mRNA in muscle cells treated with tumor necrosis factor alpha (TNF-α) and interferon gamma (IFN-γ). However, knockdown of HuR had no effect on PTGS2/COX-2 protein in these conditions [63]. Interestingly, independent study observed that HuR binds to *PTGS2* mRNA and HuR silencing decreases the expression of PTGS2/COX-2 in human colon carcinoma cells [64]. These data suggest that HuR does not directly regulate ACE2 expression in human lung fibroblasts with/without CSE, but it might regulate ACE2 expression in other conditions.

Furthermore, it is possible that ACE2 is regulated by RBPs other than HuR, such as the heterogeneous nuclear ribonucleoprotein F (hnRNP F). Overexpression of hnRNP F induces the transcription of renal *ACE2* gene, suppresses profibrotic genes (*Tgf*-*β1*, *Tgf*-*βrII*), and prevents renal fibrosis [65]. Another possibility is that ACE2 is regulated post-transcriptionally by miRNA, which are short noncoding RNAs that influence gene expression by controlling mRNA stability or by interfering with translation [66,67]. This is supported by the observation that in primary human cardiac myofibroblasts, miR-421 binds to ACE2 to down-regulate protein levels [68]. Interestingly, miR-421 is downregulated in mouse lungs exposed to cigarette smoke [69]. This raises the possibility that the upregulation of pulmonary ACE2 protein in response to cigarette smoke and/or in the context of COPD could be due the downregulation of miR-421.

In addition to cellular localization, the cleavage of HuR may also affect cellular function. In this regard, we found significant differences in HuR protein cleavage, with cleaved HuR (CP-1) being observed in smoker and COPD HLFs but not in non-smoker HLFs. This suggests that smoking itself results in HuR cleavage. To this effect, in vitro administration of CSE induced the cleavage of HuR. Cleavage of HuR protein into two fragments (CP-1, 27 kDa, and CP-2, 8 kDa) occurs in response to lethal cellular damage [42,70]. These HuR fragments are involved in apoptosis through the regulation of proapoptotic mRNAs, such as caspase-9 (*CASP9*) [42,70,71]. Given that apoptotic cell death is increased in COPD and in response to cigarette smoke as well as in the SARS-CoV-2 infection [59,72,73], it is possible that cleaved HuR expression in smoker and COPD-derived cells is driving an apoptotic phenotype and consequent lung damage in these diseases. Thus, increased cleaved HuR (CP-1) in smokers and COPD subjects may exacerbate cell death associated with SARS-CoV-2 infection, and warrants further exploration.

In summary, our work is the first to investigate the possible link between HuR and ACE2, information that is important in light of the ongoing COVID-19 pandemic. Although our data do not support a role for HuR in controlling ACE2 expression in lung fibroblasts, our study enables investigators to focus on other possible pathways, such as miRNAs, that may regulate ACE2 in the lung. We should emphasize that our conclusion, regarding the lack of a critical role for HuR in the regulation of ACE2 expression, applies only to lung fibroblasts. Further studies should be dedicated to evaluating possible role for HuR in the regulation of ACE2 expression in pulmonary and airway epithelial cells, as these cells are the port of entry of SARS-CoV-2 into the respiratory system. It should also be noted that our study demonstrates that cytoplasmic HuR is elevated in COPD lungs and that cigarette smoke induces its cleavage, a finding that may implicate HuR in the pathogenesis of both COPD and COVID-19 through the regulation of pro-inflammatory and pro-apoptotic genes that can damage the lungs. As such, our findings also point to HuR as a novel therapeutic target to combat a chronic lung disease with high morbidity and mortality, such as COPD. Further studies to mechanistically evaluate the contribution of HuR in the context of smoke-related pathologies are warranted.

## Figures and Tables

**Table 1:** Primer sequences used for RT-qPCR analysis.

Gene	Forward Primer Sequence	Reverse Primer Sequence
*ELALV1*	AAC GCC TCC TCC GGC TGG TGC	GCG GTA GCC GTT CAG GCT GGC
*ACE2-201-202*	AAC TGC TGC TCA GTC CAC CA	GAC CAT TTG TCC CCA GCA TT
*ACE2-202*	CCC AGA GCA TGC CTG ATA GA	CCC ACT TCA GAG GGT GAA CA
*S9*	CAG CTT CAT CTT GCC CTC A	CTG CTG ACG CTT GAT GAG AA

## Data Availability

All data will be made available upon reasonable request.
